# Surgery of Pancreas and Spleen

**Published:** 1894-06-30

**Authors:** 


### SURGERY OF PANCREAS AND SPLEEN.

**Pancreatic Cysts.**—Barnett records[1] a case which shows the difficulty in diagnosis of these cases, and demonstrates the inefficiency of tapping and the efficiency of incision and drainage. The patient was a farm labourer, aged twenty-four, who, after being caught between the wheels of a dray and a gatepost, became thin, weak, and dyspeptic. Nine weeks after the accident, as there was evidence of thoracic effusion as high as the eighth rib on the left side, the author aspirated the infra-axillary region, and drew off eight ounces of dark, turbid fluid. As the sequel showed, the diaphragm had been pierced, and the fluid drawn off from the abdominal cavity. About a week later a slight rounded prominence was observed in the epigastric region, which was elastic to the feel and dull on percussion; and as this continued to increase in size, it was aspirated, and subsequently other tappings became necessary. After the last tapping, a chemical examination of the fluid showed the presence of a ferment having a well-marked amylolitic action and a slight tryptic action. The abdomen was then opened, and a large cyst exposed below and to the right of the stomach. After removal of the fluid, the cavity was found to extend back to the vertebral column, and corresponded exactly to a huge cyst springing from the pancreas and distending the lesser peritoneal sac. The peritoneal coat of the cyst was attached to the parietal peritoneum, and the cyst wall stitched to the external wound, a drainage tube being left in the deepest part of the cavity. The discharge came to an end after the eighth day. Three days later the tube was removed and the fistula quickly closed up. Five months later the patient was reported as quite well and able to do heavy work as a farm labourer. Another case of pancreatic cyst (which complicated pregnancy) is reported[2] by Dr. Mayo. The course of the pregnancy was not interrupted by the operation. An abscess of the pancreas is recorded[3] by Walsh. The patient, a woman aged 47, had suffered from sharp burning pains and tenderness in the epigastrium for six months. There was an area of dulness extending from the ensiform cartilage halfway to the umbilicus, and reaching to the left costal arch. Emaciation was extreme, and she had suffered from vomiting and diarrhœa for a month. The temperature was normal. An exploratory operation was undertaken, and on passing the hand into the abdominal cavity a fluctuating mass could be felt behind the greater curvature of the stomach. This was exposed and opened, a pint of pus, together with portions of the pancreas being evacuated; at the bottom of the cavity softened remains of the body and tail of the pancreas were found and removed; this was followed by pretty free hæmorrhage, so the cavity was firmly packed with iodoform gauze. On the eighth day the stitches were removed; the fistulous track was now well-established, and the abscess cavity was reduced to about one-fourth of its former size. On the eleventh day the patient left the hospital.

**Splenic Abscess.**—Dr. Sendler has reported[4] a case spleen in a child four years old, which was cured by operation and drainage, the patient leaving the hospital thirteen days after operation. The abscess was of slow formation, and was situated in the lower portion of the spleen. At the time of operation this organ was closely adherent to the abdominal wall, the pus had passed through the capsule and would soon have found its way out. There was no predisposing cause to be found, and the only exciting cause known was a slight fall. The symptoms were very indistinct, the only one present of those which have generally been said to accompany this condition being the slight peritonitis which caused the adhesion to the abdominal parietes. The only thing that could be done was the free evacuation of the abscess.

[1] New Zealand Med. Journ., vol. 4, No. 6, Oct., 1893, and Brit. Med. Journ., Jan. 13, 1894. [2] Med. Rec., New York, Feb. 10, 1894, p. 169. [3] Brit. Med. Journ., Feb. 10, 1894, and Med. News, Dec. 30, 1893. [4] Deut. Zeit. für Chir., 1893, Bd. xxxvi., and Amer. Journ. of Med. Sciences, Mar., 1894, p. 333.

